# Anti-inflammatory actions of Pentosan polysulfate sodium in a mouse model of influenza virus A/PR8/34-induced pulmonary inflammation

**DOI:** 10.3389/fimmu.2023.1030879

**Published:** 2023-02-09

**Authors:** Ravi Krishnan, Catherine J. M. Stapledon, Helen Mostafavi, Joseph R. Freitas, Xiang Liu, Suresh Mahalingam, Ali Zaid

**Affiliations:** ^1^ Research and Development, Paradigm Biopharmaceuticals Ltd., Melbourne, VIC, Australia; ^2^ Emerging Viruses, Inflammation and Therapeutics Group, Menzies Health Institute Queensland, Griffith University, Gold Coast, QLD, Australia; ^3^ School of Pharmacy and Medical Sciences, Griffith University, Gold Coast, QLD, Australia; ^4^ Global Virus Network (GVN) Center for Excellence in Arboviruses, Griffith University, Gold Coast, QLD, Australia

**Keywords:** influenza A virus, Pentosan polysulfate sodium, acute inflammation, cytokines and chemokines, lung consolidation, lung fibrosis, chronic lung inflammation, immune cell infiltrate

## Abstract

**Introduction:**

There is an unmet medical need for effective anti-inflammatory agents for the treatment of acute and post-acute lung inflammation caused by respiratory viruses. The semi-synthetic polysaccharide, Pentosan polysulfate sodium (PPS), an inhibitor of NF-kB activation, was investigated for its systemic and local anti-inflammatory effects in a mouse model of influenza virus A/PR8/1934 (PR8 strain) mediated infection.

**Methods:**

Immunocompetent C57BL/6J mice were infected intranasally with a sublethal dose of PR8 and treated subcutaneously with 3 or 6 mg/kg PPS or vehicle. Disease was monitored and tissues were collected at the acute (8 days post-infection; dpi) or post-acute (21 dpi) phase of disease to assess the effect of PPS on PR8-induced pathology.

**Results:**

In the acute phase of PR8 infection, PPS treatment was associated with a reduction in weight loss and improvement in oxygen saturation when compared to vehicle-treated mice. Associated with these clinical improvements, PPS treatment showed a significant retention in the numbers of protective SiglecF+ resident alveolar macrophages, despite uneventful changes in pulmonary leukocyte infiltrates assessed by flow cytometry. PPS treatment in PR8- infected mice showed significant reductions systemically but not locally of the inflammatory molecules, IL-6, IFN-g, TNF-a, IL-12p70 and CCL2. In the post-acute phase of infection, PPS demonstrated a reduction in the pulmonary fibrotic biomarkers, sICAM-1 and complement factor C5b9.

**Discussion:**

The systemic and local anti-inflammatory actions of PPS may regulate acute and post-acute pulmonary inflammation and tissue remodeling mediated by PR8 infection, which warrants further investigation.

## Introduction

Acute respiratory virus infections are a significant global disease burden, as evidenced by the challenges posed by the recent SARS-CoV-2 pandemic and both previous and ongoing influenza virus endemic cycles (1–3). Severe respiratory illness caused by these viruses represents a major challenge in the clinic, and there is an unmet need for therapeutics that limit acute lung inflammation in patients by helping to manage pathology (4, 5). Mouse models of respiratory viral infection such as influenza virus and other viral and bacterial illnesses have been utilized to study the development and progression of acute lung inflammation, and to better understand how to modulate inflammatory mechanisms that exacerbate disease and lead to defective healing (6–8). PR8 is the most pathogenic species of influenza virus and comprises of a number of highly pathogenic pandemic strains, such as H1N1 and H3N2, which can cause severe illness in the elderly, children and immunocompromised individuals (9). In general, infection of airway epithelial cells results in robust pro-inflammatory cytokine production which culminates in severe inflammation, and subsequent respiratory failure (10, 11). A number of therapeutic targets have been investigated including signaling pathway inhibitors, chemokine receptor antagonists or inhibitors of neutrophil recruitment (10). Polysaccharide-based compounds have been previously shown to inhibit viral entry *in vitro*, in Respiratory syncytial virus (RSV) and SARS-CoV-2 infection (12, 13) however, there is limited information on the potential ability of PPS to ameliorate acute and/or chronic lung inflammation. Currently the management of influenza-induced illness is limited to yearly vaccination or administration of short-lasting anti-viral drugs that must be administered within 48 hours of symptom onset (14). Early corticosteroid administration is often advised in conditions such as acute respiratory distress syndrome (ARDS) to reduce lung tissue damage and mortality risk, however there is uncertainty surrounding the beneficial effects of treatment with corticosteroids in patients with severe influenza (15). ARDS is a form of severe respiratory failure where acute inflammation of the lungs results in damage of the alveolar capillary barrier, ultimately leading to leakage of oedema fluid and airway obstruction (16). Therefore, polysaccharide-based compounds that have been shown to exert some antiviral and/or anti-inflammatory functions against respiratory viruses may offer an additional treatment strategy for viral-induced ARDS.

Pentosan polysulfate sodium (PPS) is a semi-synthetic, linear polysaccharide derived from beechwood (17) that is highly sulphated. The oral formulation of PPS is an approved treatment for interstitial cystitis and bladder pain syndrome in Europe and the United States for over twenty years (18). The multiple mechanisms of action of PPS have provided the scientific rationale for repurposing the drug in other indications with unmet medical needs such as osteoarthritis (19) and alphavirus-induced arthralgia (20, 21). As PPS was demonstrated to be effective in a guinea pig model of allergen-induced rhinitis *via* its actions as an inhibitor of the Th2 cytokines IL-4, IL-5 and IL-13 (22), we extended our investigations to explore the effects of PPS in acute inflammation mediated by viral infection of the lung. The actions of PPS reported in other studies provided the rationale for its use in ARDS, which involved anti-inflammatory actions mediated *via* inhibition of NF-κB activation (23–26); inhibition of complement (C3, C3Bb and C5b9) mediated tissue injury (27); inhibition of endothelial cell activation (28); and adverse tissue remodeling by inhibiting fibroblast growth factor-2 (FGF-2) (29) and matrix metalloproteinases (MMPs), ADAMTS-4 (30–32) and elastase (33).

Based on the reported mechanisms of action of PPS, this study aimed to investigate the specific effects of subcutaneous (SC) administration of PPS on the clinical symptoms of IAV infection including weight loss & oxygen saturation and lung pathological changes associated with inflammatory cellular infiltrates and fibrosis in a sublethal PR8 mouse model using the mouse adapted H1N1 pandemic A/PR8/1934 (termed PR8) strain. In addition, the effects of PPS on inflammatory biomarkers (cytokine, chemokine and complement profiles) locally within the lungs and systemically during the acute and post-acute disease phases of PR8 infection were also investigated. The aim of this study was to evaluate the anti-inflammatory and tissue preservation properties (tissue remodeling) of PPS in a mouse model of influenza infection, and to determine the use of PPS as a potential therapeutic in both acute and post-acute phase respiratory viral infections.

## Materials and methods

### Mice, virus and infections

6–8-week-old C57BL/6J mice (purchased from Animal Resources Centre, Perth, WA) were infected intranasally with a sublethal dose of Influenza A virus (PR8) A/Puerto Rico/8/1934 (kindly donated by Prof. Carl Feng, The University of Sydney) inoculum at 300 plaque forming unit (pfu) equivalent, as determined by antibody-based endpoint dilution assay (EPDA). Mice were weighed and anesthetized with a mixture of Ketamine (80 mg/kg) and Xylazine (8 mg/kg), administered intraperitoneally in a volume of 200 μL sterile Phosphate Buffered Saline (PBS; Gibco). Mice were administered 50 μL viral inoculum (stock diluted in sterile PBS) evenly through both nostrils.

### Disease monitoring

Mice were weighed and monitored daily for signs of disease and weight loss. Weight loss was calculated as a percentage of weight change from the starting weight for each individual mouse. In accordance with AEC guidelines (Griffith University AEC# MHIQ/06/20), mice that displayed early signs of disease, including ruffled fur and lethargy, in addition to weight loss greater than 10%, were provided with wet food in the cage and hydration gel packs.

### Viral titration

Virus stock and tissue homogenates were titrated *via* EPDA. Vero E6 cells were seeded in tissue culture-treated 96-well plates until they reached 85% confluency, and serial dilutions (from neat/undiluted to 10-8) of virus inoculum were prepared in serum-free MEM. Virus dilutions were applied to the VeroE6 cell monolayer and cells were incubated for 3 days at 37°C (5% CO2). Following incubation, supernatant was removed and cells were fixed with 4% paraformaldehyde (PFA; v/v in PBS) for 5 minutes at room temperature (RT). PFA was washed off with PBS twice. Cells were permeabilized and blocked with 1% bovine serum albumin (BSA; w/v in PBS) with 0.05% Triton X-100 (v/v in PBS) for 30 minutes at RT. Blocking/permeabilising solution was removed and mouse anti-N nucleocapsid antibody (conjugated to FITC; Clone D67J; Cat#MA1-7322; Thermo Fisher Scientific) was added to the wells at a dilution of 1:2000 for 45 minutes. Cells were washed with PBS, and Hoechst 33258 (Cat#H1398; Thermo Fisher Scientific) (1:5000 of a 10 mg/mL stock in PBS) was added to the cells for 5 minutes at RT. Cells were washed and wells visualized on a Nikon Ti-E epifluorescence microscope (Nikon, Coherent Scientific, Australia) to determine the highest dilution where FITC-positive cells were present. Titer was determined as a tissue culture infectious dose titer (TCID), normalized to mL volume (and grams of tissue, where applicable).

### Treatment preparation

PPS solution was prepared fresh for each daily injection. The powdered API (bene pharmaChem GmbH & Co. KG, Germany) was weighed and taken up in 1 mL sterile PBS (1X) and solution was mixed and filtered using a 0.22 µm PVDF syringe filter. PPS was prepared at the indicated concentration (3 mg/kg or 6 mg/kg) after weighing mice and administered individually in a 200 µL volume. PPS was administered daily as indicated (from Day 0, Day 2 or Day 5) either subcutaneously in the scruff of the neck, or intraperitoneally. Vehicle (1X PBS) was administered SC or IP in a 200 µL volume as indicated.

### Tissue homogenization

Tissues were collected and placed in 500 µL cold sterile PBS in pre-weighed 2 mL SafeLock homogenization tubes (Eppendorf, Hamburg, Germany) with 6 mm sterile steel beads. Tissues were weighed and frozen at -80°C. Shortly before homogenization, tubes were partially thawed at room temperature (RT) and homogenized using a QIAGen TissueLyzer II (QIAGen) for 30 seconds at 30 repetitions per second (rps). Tubes were centrifuged at 8,000 x g for 5 minutes at 4°C and supernatants collected to perform serial dilutions used for viral titrations or tissue cytokine quantification.

### Histopathology

Lungs were collected from mice after euthanasia and placed 4% PFA (v/v in PBS) for 24 hours on a nutator at 4°C. Tissues were transferred to PBS for washing for 24 hours on a nutator at 4°C twice. Tissues were then transferred to histology cassettes and placed in 80% ethanol. Tissues were processed for paraffin embedding and non-consecutive 5 µm sections (25-30 µm apart starting at ~500 µm depth) were performed and stained with either haematoxylin and eosin (H&E) stain for cell infiltration or Masson Trichrome (MT) stain for detection of collagen. Slides were scanned using a Leica Aperio AT2 (Leica Biosystems) and images processed using QuPath software (21). For H&E-stained slides, nuclei were detected as individual objects using NucleiCount v9 algorithm on QuPath. Values generated from technical replicates of lung sections across three non-consecutive sections (25-30 µm apart) were pooled in the final analysis and graphed on GraphPad Prism (9.0). For the quantification of collagen positive fibers as an indication of tissue remodeling, a custom-designed script was devised to detect collagen fibers and quantified as a percentage of collagen-positive surface area relative to section surface area. Objects pertaining to lobes or sections of lobes were assigned using the “Simple Tissue Detection” macro, and small or torn/smeared sections were manually excluded. In addition, objects that included collagen-rich tissues such as airway, parts of trachea, bronchia and septum were manually excluded, or excised from pre-assigned objects. Using QuPath pixel classifier, specific annotations were assigned to collagen-rich structures within selected objects using the Wand tool. Non-collagen pixel objects were assigned specific annotations, and background was assigned as either “Negative” or “Ignore” annotation. Using the “Train Pixel Classifier” algorithm, the detection of blue, collagen-denoting pixels was verified on “Normal Resolution”. Pixel classifier algorithm was further trained to eliminate false or approximate collagen/blue pixel detection (5-7 iterations). A final validation was performed using “High” or “Very high” resolution, and classifier further applied to a random selection of images (2, 3). Upon final validation, the algorithm was input into a Script Editor to run multi-file batches.

### Pathology scoring

Additional assessment of cellular inflammation (H&E-stained slides) and tissue remodeling-associated collagen (Masson Trichrome-stained slides) and fibrosis-associated morphological changes was performed by an expert histopathologist who was blinded to the identity of the sections. A validated pathology scoring system (22, 23) was used to assign scores according to a scale from 0 to 5 where: 0 = no change; 1 = minimal change (or possibly non-specific background or appearance compounded by lung collapse, i.e., not inflated); 2 = mild change; 3 = moderate change; 4 = severe change in <50% of lung lobe; 5 = severe change in >50% of lung lobe. Data was de-identified and transferred to GraphPad Prism (9.0) for plotting and statistical analysis. Technical replicates of lung sections across three non-consecutive sections (25-30 µm apart) were pooled in the final analysis and graphed on GraphPad Prism (9.0).

### Oxygen saturation

SpO_2_ measurements were performed using a StarrLife MouseOx sensor pulse oximetry measurement instrument. Briefly, mice were anesthetized using 1-2% isoflurane (in a nebulized air mixture) and hind legs shaved and depilated using a depilatory cream (Veet). Pulse oximetry was measured using a thigh sensor, and values were recorded for each mouse.

### Immunofluorescence microscopy

Lung tissues were harvested and fixed in 4% paraformaldehyde overnight. The tissues were subsequently washed in PBS and dehydrated in in 30% sucrose overnight. The tissues were embedded in optimal-cutting-temperature (OCT) compound (Sakura Finetek) and then frozen at -80°C. Tissue blocks were cut into 20 μm-thick sections and permeabilized in cold acetone. The sections were blocked with serum-free protein block (DAKO) and immunolabelled with antibodies against mouse CD169 (AbD Serotec), Gr-1 (BD Biosciences), and Collagen Type IV (Abcam). Nuclei were stained with 0.025 mg/ml Hoechst33258 (Thermo Fisher Scientific). The slides were mounted with ProLong Gold antifade (Thermo Fisher Scientific). Images were acquired using a confocal microscope (Olympus FV3000) using a 20x (0.95 NA) objective and processed using Imaris (v9.5; Bitplane).

### Flow cytometry

Lungs were processed for flow cytometry analysis using a protocol adapted from Liu et al. (24), shown in **Supplemental Figure 3**. Briefly, mice were euthanized by ketamine/xylazine overdose, and perfused intracardially with ice-cold 1x Phosphate Buffered Saline (PBS; Gibco). Experimental procedures were performed under aseptic conditions and within a BSC-II cabinet. Lungs (left lobe) were collected in ice-cold PBS until processing for flow cytometry. Lungs were cut into small pieces using sterile scissors in 1 mL Digestion Solution (3mg/mL Type IV Collagenase [Worthington Co.] with 10 µg/mL DNAse I [Sigma Aldrich] in RPMI [Gibco]). Dissected lungs were then added to 2mL digestion solution and incubated for 1 hour at 37°C. Samples were added to 3 mL RPMI + 10% FCS and pipetted vigorously for 10 seconds using a Pasteur pipette, then filtered through a 70 µm cell strainer. The strainer was further rinsed with 5 mL RPMI, and samples centrifuged at 375 x g for 5 minutes at 4°C. Supernatant was decanted and pellets were resuspended in ACK lysis buffer (0.15M NH_4_Cl; 0.01M KHCO_3_; 0.1mM Na_2_EDTA; pH 7.2) for 5 minutes at RT to lyse red blood cells. Lysis was stopped with 10 mL PBS, followed by centrifugation at 375 x g for 5 minutes at 4°C. Single cells were resuspended in FACS buffer (PBS with 1% bovine serum albumin [BSA; Sigma Aldrich] and 0.5 mM EDTA) and placed on ice. For antibody staining, cells were first incubated with anti-mouse CD16/32 (Fc block; BD Biosciences) antibody for 15 minutes at 4°C before addition of fluorochrome-conjugated antibodies. Cells were immunolabelled with the following anti-mouse antibodies: CD45-BUV805 (BD Biosciences), CD11b-BV711 (BD Biosciences), CD64-BV650 (BD Biosciences), CD8-AlexaFluor594 (Biolegend), CD4-BV510 (BD Biosciences), CD3-APC (eBiosciences), SiglecF-PE (eBiosciences), Ly6C-PeCy7 (eBiosciences), Ly6G-BV786 (BD Biosciences), CCR2-BUV496 (BD Biosciences) and LIVE/DEAD NIR (Thermo Fisher Scientific) cell viability dye. Antibody staining was performed for 40 minutes in the dark at 4°C and cells were subsequently washed with ice-cold FACS buffer. Cells were fixed in 4% paraformaldehyde (v/v in PBS) for 5 min at 4°C, washed twice with FACS buffer, filtered through a 30 µm cell strainer and resuspended in 200 µL PBS. Counting beads (Sphero Calibration beads, BD Biosciences) were added to the samples before acquisition on a BD LSR Fortessa X20 flow cytometer, and data analyzed using FlowJo v10.7 (TreeStar Inc.).

### Collection of tissues and processing for viral titers and cytokine/complement analysis

Mice were euthanized by lethal overdose with ketamine/xylazine prior to cervical dislocation. The thoracic cage was dissected, and mice were exsanguinated *via* an excision of the right atrium. Lungs were collected in pre-weighed 2 mL homogenization tubes containing 500 µL ice-cold PBS and a 6 mm diameter stainless steel bead (QIAGEN; Cat#69989). Samples were kept on ice during the procedure, weighed and transferred to -80°C for storage. Prior to homogenization, samples were allowed to partially thaw at 4°C for 2 hours. Samples were loaded on a TissueLyzer II (QIAGEN) and homogenized for 30 seconds at 20 repetitions per second. Samples were then centrifuged at 5000 x g for 5 minutes at 4°C, and supernatant collected for either viral titration (as per plaque assay protocol) or cytokine/complement protein analysis. Cytokine analysis was performed using a multiplex cytokine bead array (CBA) Mouse Inflammation kit (BD Biosciences; Cat#552364) which enables simultaneous detection of pro-inflammatory cytokines CCL2, IL-6, IFN-γ, IL-12p70, IL-10 and TNF-α. Samples were processed as per manufacturer’s instructions and acquired on a BD LSR Fortessa flow cytometer. Data was exported and analyzed using the FCAP Array v3.0 software (BD Biosciences). Complement proteins were quantified using single ELISA kits (Novus Biologicals Mouse Complement C3a ELISA kit, Cat#NBP2-70037; Thermo Fisher Mouse Complement C5a ELISA kit, Cat#EHMC; Hycult Biotech Mouse Complement C3b ELISA kit, Cat#HK216-01; Biomatik mouse Terminal Complement Complex C5b9 (Cat#EKU10320); Cusabio mouse Factor B (Complement Factor Bb; CF-Bb) Cat#CSB-E04695m); R&D Systems mouse soluble ICAM-1 (sICAM-1) Quantikine Cat#MIC100) according to manufacturer’s instructions. Optical density values (450nm and 570nm) were read on a POLARStar Omega plate reader (BMG Labtech) and data exported to .csv files using the manufacturer’s proprietary software.

### Statistical analyses

Statistical analyses were performed using GraphPad Prism v9. Comparisons between groups were performed using a one-way ANOVA with a Kruskal-Wallis test, and two-variable comparisons were performed using a two-way ANOVA. Datasets were assessed for normality (normal or non-normal distribution), and statistical tests were applied accordingly. Where applicable, an outlier test (ROUT; Q=5%) was performed to exclude outliers from the data set. Statistically significant differences are shown on plots as numerical p values (adjusted) or using asterisks (i.e., ns. Not significant; *p<0.05; **p<0.01; ***p<0.005; ****p<0.001).

## Results

### PPS ameliorates acute disease in C57BL/6J mice infected with PR8

**Figure 1:**
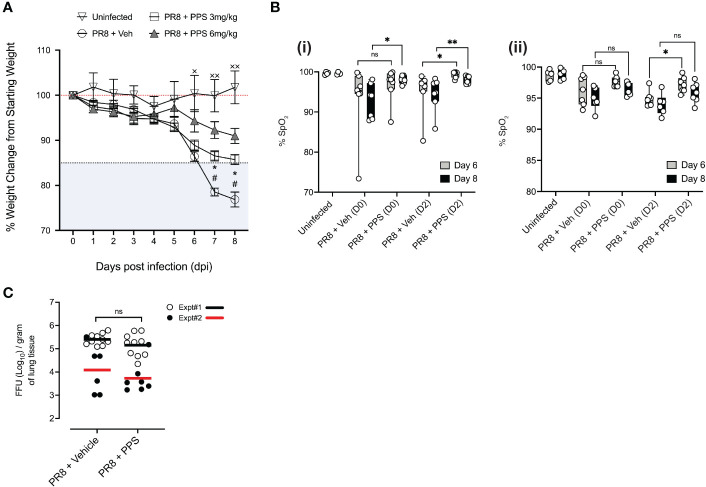
Effects of daily PPS treatment on sublethal PR8-induced disease assessments. Mice were treated daily with 3 mg/kg or 6 mg/kg PPS, or vehicle, starting from Day 0 to Day 7 post-infection. **(A)** Weight loss in uninfected mice, PR8-infected mice treated with 3 mg/kg or 6 mg/kg PPS or with vehicle SC *^#^p<0.05* for PR8 + Veh compared with PR8 + PPS 3 mg/kg; *p<0.05 for PR8 + Veh compared with PR8 + PPS 6 mg/kg; ^**×**^ *p<0.05*, ^**××**^ *p<0.01* for PR8 + Veh compared with Uninfected. Statistically significant differences were assessed by two-way ANOVA. **(B)** Pulse oximetry was measured at Day 6 and Day 8 post-infection across two independent experiments, shown in (i) and (ii). Mice were infected with PR8 and treated with PPS 3 mg/kg at Day 0 or Day 2 post-infection. Statistically significant differences were assessed by one-way ANOVA; *p* values shown on plots; *p<0.05; **p<0.01; ns, not significant. ((**i**) and (ii) n=7 mice per group; n=4 mice in the uninfected group). **(C)** Effect of PPS treatment on viral titres in the lungs of mice infected with PR8. PR8-infected mice treated with vehicle SC (PR8 + Vehicle), PR8-infected mice treated with PPS SC (PR8 + PPS); n=7-10 mice per group. Data shown from two independent experiments, denoted Expt#1 and Expt#2. Mean values for each experiment depicted by black (Expt#1) and red (Expt#2) lines on plots. Statistically significant differences were assessed on pooled data by student’s t-test; *p* values shown on plots. ns: not significant.

Sub-lethal infection with the PR8 strain of influenza A virus causes ARDS in immunocompetent C57BL/6J mice, which is characterized by sharp weight loss generally observed between 5- and 7-days post-infection (dpi) (7, 8). To determine whether PPS was effective in reducing PR8-induced disease severity, mice were treated with either 3 or 6 mg/kg PPS subcutaneously (SC) at 2 hours post-PR8 infection with a sublethal dose [300 plaque forming units (pfu)] of intranasal PR8. For our acute phase study, vehicle (PBS), and PPS were administered every 24 hours from 0- 7 dpi. For our subsequent post-acute phase study, we monitored mice until 21 dpi. Mice infected with PR8 and administered vehicle (SC) displayed severe disease, as evidenced by the sharp weight loss starting from 6 dpi, reaching 10-15% weight loss from starting bodyweight (**Figure 1A**). Weight loss in mice treated with 3 mg/kg SC PPS was significantly reduced compared to that observed in vehicle-treated mice at 7 and 8 dpi (p<0.05), with the average weight loss approaching the 15% endpoint threshold at 8 dpi. In contrast, mice treated with 6 mg/kg SC PPS displayed less pronounced weight loss at 7 and 8 dpi compared to mice treated with 3 mg/kg PPS (**Figure 1A**). Based on previous reports of systemic effect of PPS (24), we also investigated the effect of PPS administered intraperitoneally (IP) in mice infected with PR8. Intraperitoneal administration of PPS led to a statistically significant reduction in weight loss at 6 (p<0.01) and 7 dpi, (p<0.005), but not 8 dpi (p=0.0809), in PR8-infected mice when compared to vehicle-treated mice (**Supplemental Figure 1**). As the reduction in weight loss in the IP group was not as pronounced as what was observed in the SC group, the subcutaneous route was chosen for all subsequent experiments.

To better understand the physiological effect of PPS in the context of acute respiratory disease in PR8-infected mice, we measured blood oxygen saturation (SpO_2_) at 6 and 8 dpi in mice treated with 3 mg/kg PPS, across two independent experiments and tested whether the delayed administration of 3 mg/kg PPS, from 2 dpi, may exert a therapeutic effect in infected mice (**Figure 1B**; i and ii). In the first experiment (**Figure 1B**; i), infected mice treated with PPS from Day 0 demonstrated significantly improved oxygen saturation at 8 dpi (**Figure 1B**; i). Although the differences at 6 dpi were not statistically significant, SpO_2_ was higher in PPS-treated mice compared to vehicle-treated mice (**Figure 1B**; i). Mice treated with PPS from Day 2 showed a statistically significant increase in SpO_2_ at both 6 and 8 dpi, when compared with infected mice administered vehicle. In the repeat experiment (**Figure 1B**; ii), only mice treated with PPS from Day 2 demonstrated a significant increase in SpO_2_ when compared to vehicle-treated mice at 6 dpi. While not statistically significant, there was an improvement in SpO_2_ in mice treated with PPS from Day 2 compared with mice treated with vehicle (**Figure 1B**; ii).

### PPS does not affect viral clearance in lungs

Reducing tissue inflammation during virus-induced disease without enhancing viral replication is an important characteristic of any immune-modulatory compound in the context of pre-clinical assessment. To investigate the effects of PR8 infection and PPS as a treatment option, lung viral titers at 8 dpi were measured. Over the course of two independent experiments (Expt#1 and Expt#2), there were no statistically significant differences in viral titers between the lungs of mice treated with 3 mg/kg PPS and those that received vehicle (**Figure 1C**), indicating that reduced disease severity seen in PPS-treated mice at 8 dpi is not associated with a reduction in viral titers, nor does it confer an advantage for viral replication.

### Histological assessment of lung pathology in acute PR8-induced disease

**Figure 2:**
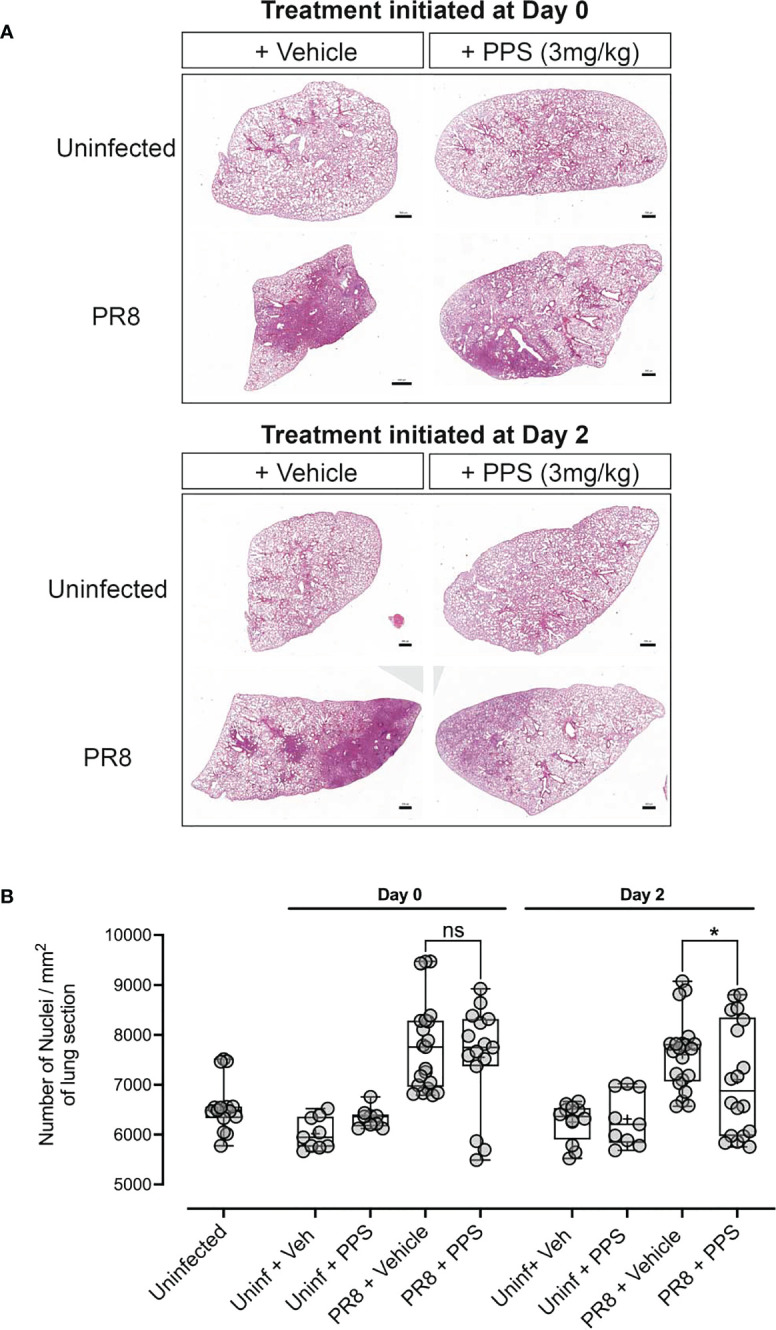
Histological analysis of lungs in PR8-infected mice treated with PPS. **(A)** H&E staining of lung sections and nuclei counts from uninfected mice and mice infected with PR8 and treated with 3 mg/kg PPS from Day 0 or Day 2 post-infection. **(B)** Quantification of cellular infiltrates in the lungs [from data shown in **(A)**] of PR8-infected mice treated with 3 mg/kg PPS. n=7 for all PR8 groups, n=3 for Uninfected + PPS, n=3 for Uninfected + Veh, n=5 for Uninfected). Each data point represents nuclei counts calculated on individually detected objects (using automated software object detection) in triplicate sections for each group. Statistically significant differences were assessed by one-way ANOVA with a Sidak post-test. *p<0.05 shown in plots. (ns, not significant). Box-whiskers plot show all data points (max/min), standard error, median (line in box) and mean (‘+’ in box). Each data point represents one pathological score assigned to a lobe assessed in triplicate sections for each group.

As the data suggested that PPS treatment could dampen severe PR8-induced disease without affecting lung viral titers, the impact of PPS on lung infiltration and tissue morphology was investigated histologically *via* haematoxylin & eosin (H&E) staining of lung sections (**Figure 2A**). Lung alveolar spacing appeared reduced in PR8-infected, vehicle-treated mice in both Day 0 and Day 2 treatment groups, and consolidation of the parenchyma - a pathological feature of respiratory infections that leads to an increase in density of the lung parenchyma (34) - was increased compared to PPS-treated mice. Upon further inspection, alveolar density appeared less pronounced in the Day 2 PPS cohort when compared to the Day 0 cohort. We did not observe any statistically significant reduction (p=0.4708) in leukocyte infiltrates in the lungs of mice treated from Day 0 with 3 mg/kg SC PPS compared to vehicle-treated mice, but PPS administered from Day 2 was associated with a significant reduction (p=0.0445) in infiltrates compared to infected control mice (**Figure 2B**). Quantitation of lung sections from uninfected control mice (uninfected + vehicle and uninfected + PPS) showed counts similar to those observed in uninfected, untreated mice (**Figure 2B**).

### PPS treatment selectively alters the lung immune cell profile

**Figure 3:**
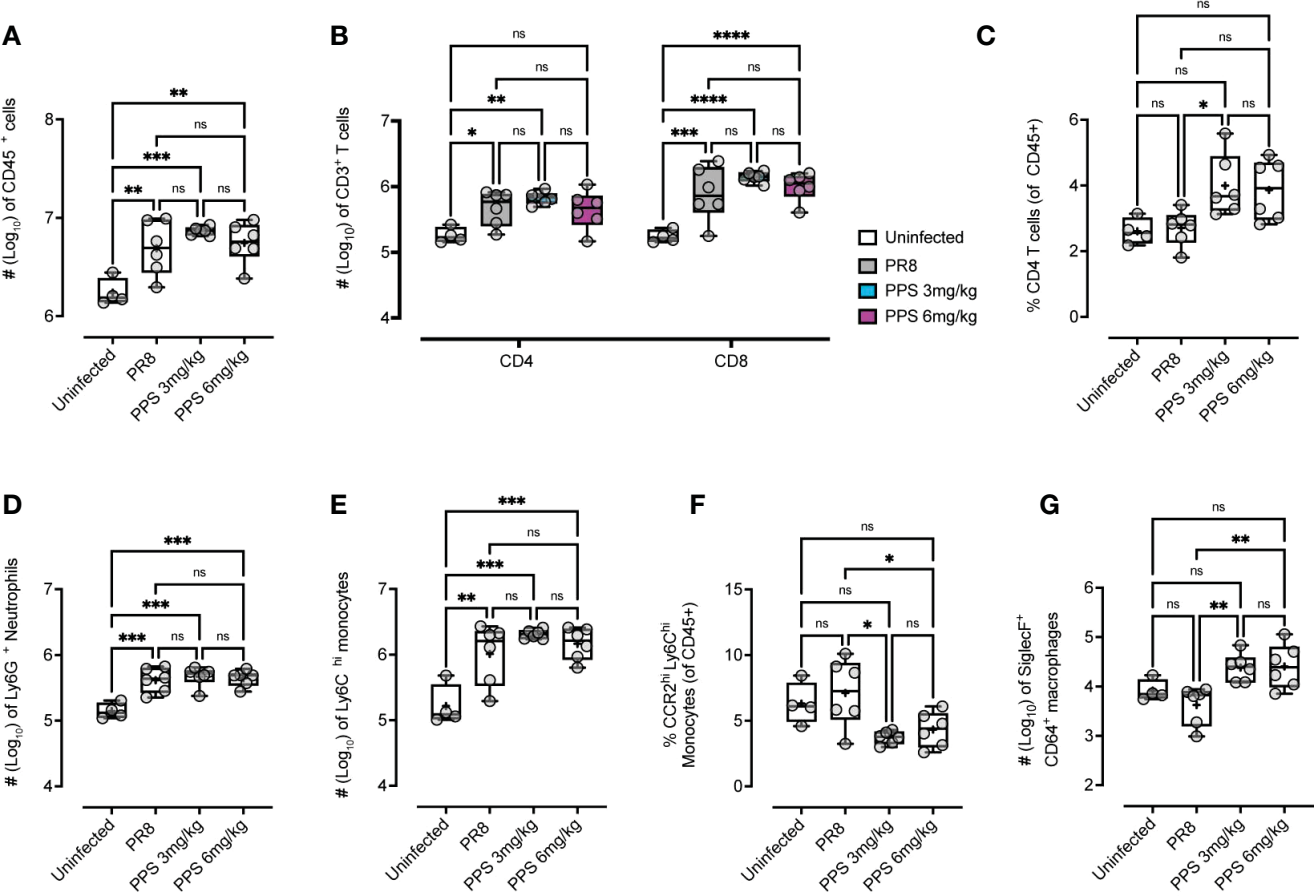
Flow cytometry analysis of changes in the number of cellular infiltrates in the lungs of mice infected with PR8 and treated with PPS (3 mg/kg and 6 mg/kg, SC). Enumeration of **(A)** total live CD45^+^ cells, **(B)** CD3^+^ CD4^+^ and CD8^+^ T cells, **(C)** the percentage of CD4^+^ T cells (of total CD45^+^ leukocytes), **(D)** Ly6G^+^ neutrophils, **(E)** Ly6C^hi^CD11b^+^ inflammatory monocytes (IM), **(F)** the percentage of CCR2^hi^ inflammatory monocytes cells (of total CD45^+^ leukocytes) and **(G)** SiglecF^+^ CD64^+^ alveolar macrophages. Data shown as either Log_10_ number of cells (normalized to tissue weights) or % of cell subset of total CD45^+^ cells. Statistically significant differences were assessed by one-way ANOVA with a Tukey post-test (ns, not significant; *p<0.05; **p<0.01; ***p<0.005; ****p<0.001). Box-whiskers plot show all data points (max/min), standard error, median (line in box) and mean (‘+’ in box). Sample size: n=6 mice per group; n=4 mice for uninfected group.

Next, the effect of PPS treatment on immune cell infiltration in the lungs of PR8-infected mice was assessed by flow cytometry. Data in **Figure 1A** indicated that while mice treated with 3 mg/kg PPS showed reduced weight loss compared to vehicle-treated mice, this effect was enhanced in mice treated with 6 mg/kg PPS, pointing towards a potential dose-dependent effect of PPS in the context of acute PR8-induced disease. To determine whether this effect could be explained by a difference in host immune responses, lungs were collected at 8 dpi and processed for immune cell analysis. The number of CD45+ cells was significantly higher in the lungs of PR8-infected mice compared to uninfected mice, but there were no statistically significant differences in the overall number of CD45+ cells between PR8-infected vehicle-treated mice and mice treated with SC PPS at either 3 or 6 mg/kg (**Figure 3A**). While infection with PR8 was associated with an increase in the number of CD4+ and CD8+ T cells in the lungs, there were no statistically significant differences between PR8-infected mice treated with vehicle and mice treated with PPS at either 3 or 6 mg/kg (**Figure 3B**). While absolute CD4+ T cell numbers in the lungs were not significantly different in the lungs of PPS-treated mice, we observed that the percentage of CD4+ T cells (of all CD45+ cells) was significantly higher in the lungs of mice treated with 3 mg/kg PPS compared with PR8-infected mice that received vehicle (**Figure 3C**). Despite not being statistically significant, PPS-treated mice (6 mg/kg) exhibited a higher percentage of CD4+ T cells than mice treated with vehicle. (**Figure 3C**).

**Figure 4:**
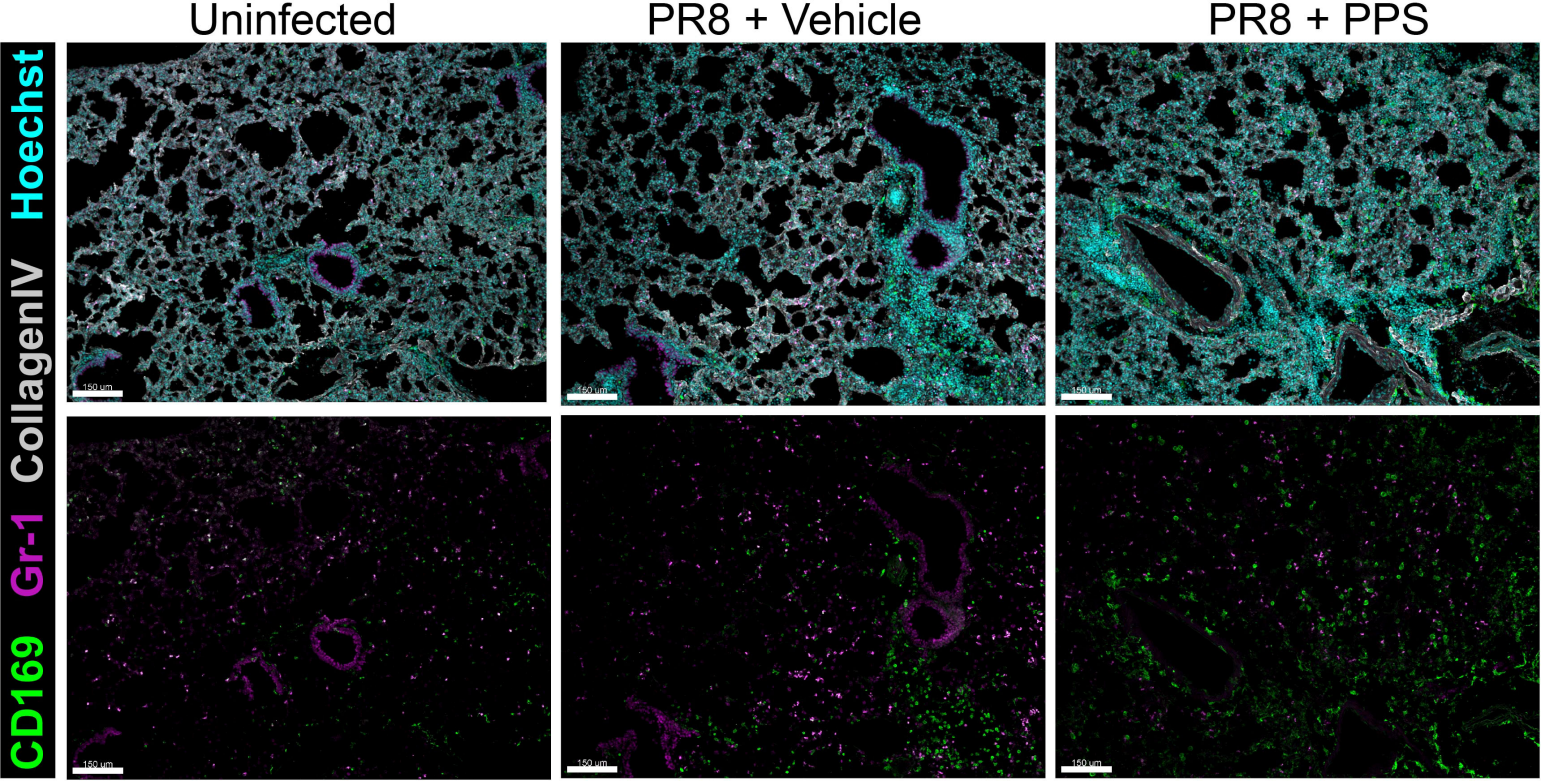
Immunofluorescence microscopy of lungs in PR8-infected mice treated with 6 mg/kg SC PPS. Representative confocal immunofluorescence micrographs of lung cryosections from uninfected mice, PR8-infected mice (vehicle-treated; PR8 + Vehicle) and PR8-infected mice treated with 6 mg/kg PPS (PR8 + PPS). Sections were immunolabelled with anti-mouse CD169, anti-mouse Gr-1 and anti-mouse Collagen Type IV. Nuclei were labelled using Hoechst (33258) and images acquired as z-stacks by confocal microscopy. Images shown as maximum intensity projections. Upper panels show merged channels. Scale bars = 150 μm.

In the myeloid compartment, there were no significant differences in the number of Ly6G+ neutrophils (**Figure 3D**), or Ly6C^hi^ inflammatory monocytes (**Figure 3E**) between vehicle and PPS-treated groups, however, within CD45+ immune cells the subset of CCR2^hi^ inflammatory monocytes was significantly reduced in the lungs of mice treated with PPS at both 3 and 6 mg/kg, when compared to vehicle-treated mice (**Figure 3F**). Interestingly, the number of SiglecF+ alveolar macrophages, a subset of lung-resident macrophages implicated in local immune responses against respiratory pathogens, and associated with protection against viral infection, was significantly higher in the lungs of mice treated with PPS at both 3 and 6 mg/kg, compared to vehicle-treated mice (**Figure 3G**). This was also observed *in situ* using confocal immunofluorescence microscopy of lung cryosections (**Figure 4**) collected at 8 dpi, which outlines the distribution of Gr-1-expressing monocytes and neutrophils, and CD169+ lung-resident alveolar macrophages in the lung tissue. Alveolar spacing in uninfected mice is characterized by low-density parenchyma, and sparse distribution of Gr-1+ and CD169+ cells. In contrast, PR8-infected lungs of mice treated with vehicle display areas of consolidation with dense clusters of immune cells, characteristic of large infiltration of circulating cells (neutrophils, monocytes) and alveolar macrophages. Interestingly, we observed a broader distribution of CD169+ alveolar macrophages - and conversely, a more restricted distribution of Gr-1+ cells across the parenchyma at 8 dpi in mice treated with PPS compared to vehicle-treated mice (**Figure 4**).

### Effect of PPS on complement proteins and pro-inflammatory cytokines

**Figure 5:**
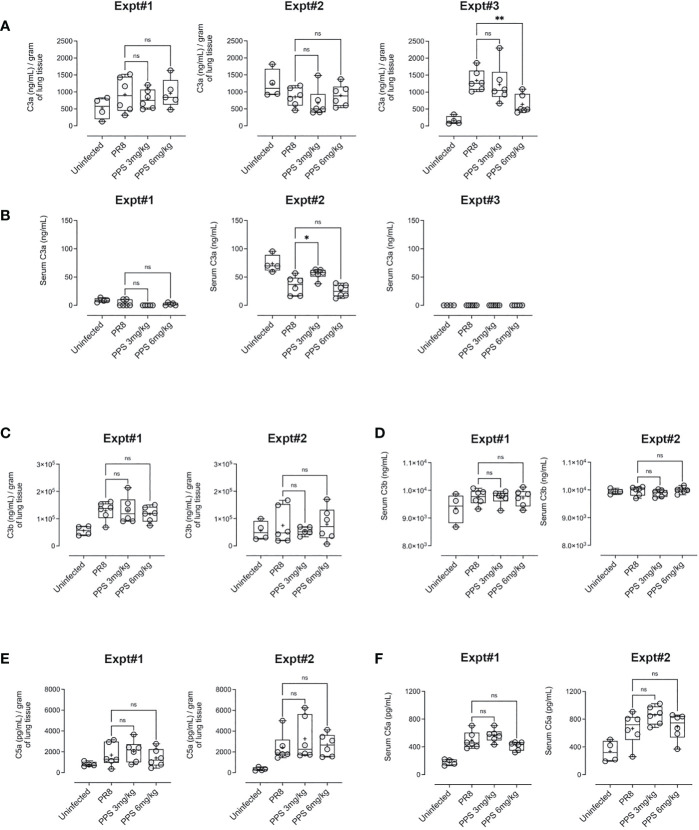
Complement protein measurement in the lungs and serum of PR8-infected mice treated with PPS. **(A)** Changes in lung levels of C3a, C3b and C5a measured by ELISA. Values normalised to tissue weights. **(B)** Changes in serum concentration of C3a, C3b and C5a measured by ELISA. Box-whiskers plot show all data points (max/min), standard error, median (line in box) and mean (‘+’ in box). Statistically significant differences were assessed by one-way ANOVA with a Sidak post-test (ns, not significant);. Data shown is from three individual, independent experiments denoted Expt#1, Expt#2 and Expt#3 (for C3a in 5a, 5b; total n=12-18 mice) and two independent experiments denoted Expt#1 and Expt#2 (for C3b in **(C, D)** and C5a in **(E, F)** total n= 7-12 mice).

**Figure 6:**
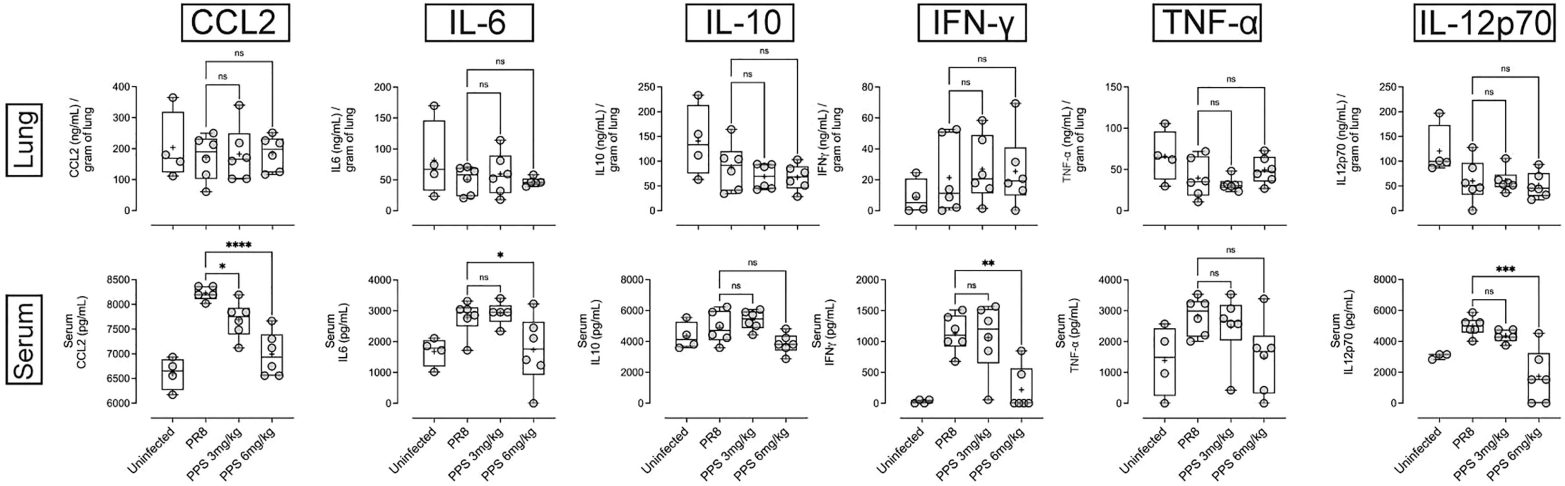
Pro-inflammatory cytokine/chemokine measurement in the lungs and serum of PR8-infected mice treated with PPS. Changes in lung and serum levels of CCL2, IL-6, IL-12p70, IL-10, IFN-γ and TNF-α measured by ELISA in Experiment 1. Values for lung concentrations were normalised to tissue weights. Values for serum concentrations are shown as per mL of serum. Box-whiskers plot show all data points (max/min), standard error, median (line in box) and mean (‘+’ in box). Statistically significant differences were assessed by one-way ANOVA with a Sidak post-test (*p<0.05; **p<0.01; ***p<0.001; ****p<0.0001; ns: not significant). (n=6 mice per group per experiment; Data shown is one of 3 independent experiments).

PPS has been shown to interfere with components of the complement pathway, including C3a and C5a (27, 33), which are likely to contribute to airway inflammation in acute PR8 infection (29). Therefore, we determined lung and serum levels of C3a, C3b and C5a by ELISA at 8 dpi in PR8-infected mice treated with vehicle, 3 or 6 mg/kg PPS. Across three independent experiments, we observed variable outcomes in both serum and lungs. While one out of three experiments showed that lung C3a levels were significantly reduced in mice treated with 6 mg/kg PPS (**Figure 5A**), this difference was not reflected in the serum of mice from the same experiment (**Figure 5B**). Likewise, we did not observe any statistically significant differences in C3b in either the lungs (**Figure 5C**) or serum (**Figure 5D**), as was the case for lung (**Figure 5E**) and serum (**Figure 5F**) C5a levels between vehicle- and PPS-treated groups. Lung and serum levels of pro-inflammatory cytokines and chemokines (CCL2, IL-6, IL-10, IL-12p70, IFN-γ and TNF-α) were also measured in three independent experiments (**Figure 6**). In one experiments (Experiment 1, shown in **Figure 6**), serum levels of CCL2 were significantly reduced in PPS-treated mice (3 and 6 mg/kg), as were serum levels of IL-6 (6 mg/kg), IFN-γ (6 mg/kg) and IL-12p70 (6 mg/kg) compared with vehicle-treated mice (**Figure 6**). It is worth noting that Experiment 1 showed a more relevant baseline as cytokine levels in the control (uninfected) group were lower compared to those detected in infected groups, whereas this baseline cytokine readout in control mice was higher in Experiments 2 and 3 (Expt#2 and Expt#3; shown in **Supplemental Figure 2**). Overall, except for the significant reduction in IL-12p70 (**Supplemental Figure 2**; Expt#3) in the lungs of mice treated with 3 and 6 mg/kg PPS, we did not observe any significant differences in lung tissue cytokine/chemokine concentration following PPS treatment compared to PR8-infected mice treated with vehicle.

### Effect of PPS on post-acute PR8-induced lung pathology

#### i) Body weight changes during post-acute phase of PR8 infection

**Figure 7:**
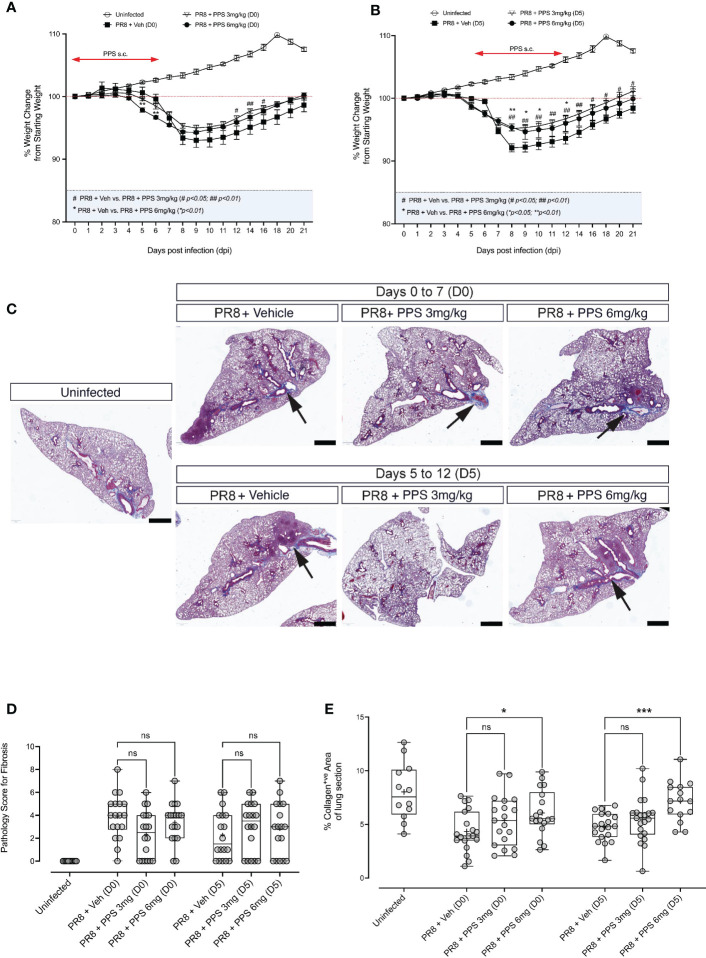
Histological analysis of post-acute (21 dpi) lung fibrosis and tissue remodeling. **(A)** Weight loss curves for PR8-infected mice administered 3 or 6 mg/kg PPS at Day 0 or **(B)** Day 5 post-infection. Mice were monitored and weighed daily until 12 dpi and every second day from 12 dpi until Day 21 dpi. Statistically significant differences in weight loss compared to vehicle-treated, PR8-infected mice shown as # (comparing PR8+Veh and PR8 + PPS 3mg/kg) and * (comparing PR8+Veh and PR8 + PPS 6mg/kg). Mean values for each dpi were analysed by two-way ANOVA with Dunnett’s post-test. ^*/#^p<0.05; ^**/##^p<0.01. Sample size: n=6 mice per group; n=4 mice for uninfected group. **(C)** Representative micrographs of Masson-Trichrome (MT) stained lung sections from mice infected with PR8 and treated with SC PPS at 3 mg/kg or 6 mg/kg, with treatment starting at Day 0 or Day 5. Black arrows point to the blue MT staining which indicates areas of collagen deposition; red staining indicates cell nuclei. **(D)** Pathology scoring of lung sections shown in **(A, B)**. Sections were assessed according to a pathology scoring matrix, where 0 = no change; 1 = minimal change (or possibly non-specific background or appearance compounded by lung collapse, i.e., not inflated); 2 = mild change; 3 = moderate change; 4 = severe change in <50% of lung lobe; 5 = severe change in >50% of lung lobe. n=6 per group; n=4 for uninfected group. Number of slides per group: n=3 per group. Box-whiskers plot show all data points (max/min), standard error, median (line in box) and mean (‘+’ in box). Statistically significant differences between groups were determined using a non-parametric one-way ANOVA (Kruskal-Wallis), and *p* values shown on the plots. **(E)** Automated quantification of collagen deposit and fibrosis in MT-stained lung sections in mice infected with PR8 and treated with SC PPS at 3 mg/kg or 6 mg/kg, with treatment starting at Day 0 or Day 5. Statistically significant differences between groups were determined using a non-parametric one-way ANOVA (Kruskal-Wallis). P values shown on plots: *p<0.05; **p<0.01; ***p<0.005; ns, not significant. Individual data points represent single value of % collagen signal coverage per lung section. Box-whiskers plot show all data points (max/min), standard error, median (line in box) and mean (‘+’ in box). (n=6 per group; n=4 for uninfected group. Number of slides per group: n=3 per group).

The development of chronic lung disease following severe PR8 infection, in particular lung consolidation and/or fibrosis that result from lung tissue damage and remodeling are clinically important, because they may give rise to chronic manifestations. As our data indicates that PPS treatment can reduce acute disease in PR8-infected mice, we asked whether this was associated with lung consolidation in the post-acute phase. In addition, we sought to determine whether SC PPS administered therapeutically during peak PR8 infection and disease (5 - 12 dpi) could result in reduced post-acute lung pathology, i.e., lung consolidation. PR8-infected mice were treated with vehicle or PPS, at 3 or 6 mg/kg, from 0 dpi to 7 dpi, or from 5 dpi to 12 dpi. Over 21 days of infection, we observed a significant reduction in weight loss in mice treated with PPS from Day 0 (**Figure 7A**), but this was confined to early (i.e., 5 and 6 dpi) time points for mice treated with 6 mg/kg PPS, and later (12, 14 and 16 dpi) time points for mice treated with 3 mg/kg PPS. Interestingly, mice treated therapeutically with PPS from Day 5 displayed more consistent reduction in weight loss (**Figure 7B**), as mice treated with 3 mg/kg PPS showed a significant reduction between 8 and 21 dpi, and mice treated with 6 mg/kg showing improved weight gain at 8, 9, 10 and 12 dpi.

#### ii) Effect of PPS on lung histopathology

At 21 dpi, mice were euthanized and lung tissues were processed for histological analysis and stained with Masson’s Trichrome (MT) to determine the extent of collagen fibre deposition, a readout for lung tissue remodeling. Histological sections outline the deposition of collagen in tissues, which appears blue against a pink (eosin) counterstain (**Figure 7C**). Areas of lung alveolar space consolidation and more intense collagen stain was observed, however no clear visual differences between PPS- and vehicle-treated mice were observed. Therefore, lung tissue consolidation was assessed *via* two distinct approaches: 1) blinded, qualitative pathology scoring, and 2) automated/computer assisted quantification of tissue collagen. While some reduction in pathology score was observed in lungs of mice treated with PPS from Day 0 (D0; 3 and 6 mg/kg), these differences were not statistically significant (**Figure 7D**). Likewise, sections from mice treated from Day 5 (D5; 3 and 6 mg/kg) showed no statistically significant differences in pathology score compared to vehicle-treated mice (**Figure 7D**). Similarly, our analysis of collagen fiber deposition using a pixel classification algorithm did not reveal any significant reductions in collagen signal in MT-stained sections (**Figure 7E**).

#### iii) Effect of PPS on fibrotic biomarkers in PR8 infected lungs in the post-acute phase

**Figure 8:**
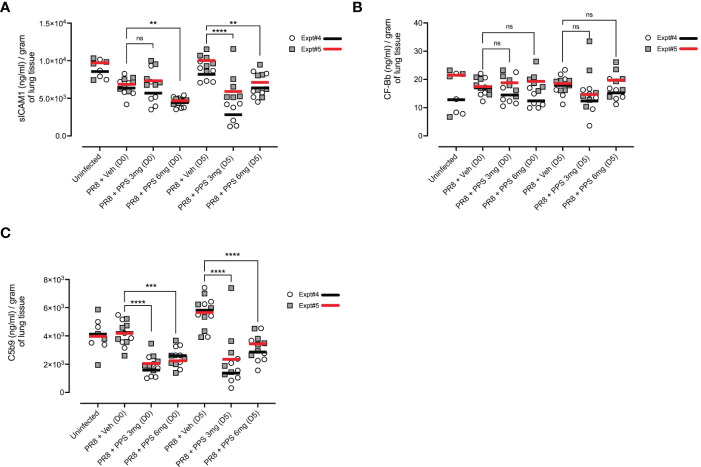
Measurement of pro-fibrotic and remodeling factors in the lungs of PR8-infected mice treated with PPS at 21 dpi. Mice were infected with PR8 and treated with 3 or 6 mg/kg PPS at Day 0 or Day 5 post-infection, daily for 7 days. Lung concentration of **(A)** sICAM-1 **(B)** Complement Factor Bb (CF-Bb) and **(C)** C5b9. All values normalised to tissue weights. Data shown from two independent experiments, denoted Expt#4 and Expt#5. Mean values for each experiment shown by black (Expt#4) and red (Expt#5) line on plot. Statistically significant differences were assessed on pooled data by a Student’s t-test. Statistically significant differences of pooled values were assessed by one-way ANOVA with a Sidak post-test (ns, not significant; **p<0.01; ***p<0.005; ****p<0.001). (sample size: n=4-6 mice/group).

We next asked whether cytokine and signaling pathways associated with fibrosis in the post-acute phase were impacted by PPS treatment during acute infection. As soluble ICAM-1 (sICAM-1), and the C5 complement pathway have been shown to be associated with lung fibrosis (35–38), we measured lung tissue concentrations of sICAM-1 and complement proteins, Complement Factor Bb (CF-Bb) and C5b9 by ELISA across two independent experiments (shown as Expt#4 and Expt#5 in **Figure 8**). In PR8-infected mice treated with 6 mg/kg PPS from both Day 0 and Day 5, sICAM-1 levels at 21 dpi were significantly reduced compared with vehicle-treated mice (**Figure 8A**). Levels of CF-Bb were not affected by PPS treatment (**Figure 8B**) at either concentration or dose timing. Finally, levels of C5b9 were significantly reduced (**Figure 8C**) in the lungs of mice treated with PPS at either 3 or 6 mg/kg doses, and in both Day 0 and Day 5 treatment groups.

## Discussion

In this study, a well-established experimental model of PR8 mediated pulmonary infection was examined to determine whether PPS, a semi-synthetic polysaccharide could favorably alter the outcome of PR8-induced disease in mice. PPS has been shown to interfere with NF-κB signaling, which consequently downregulates cytokines and complement proteins that contribute to potent inflammatory responses and tissue damage (26, 39, 40). In addition, the use of PPS in pre-clinical models of inflammatory viral disease, such as viral arthritis caused by arthritogenic alphaviruses, suggests that this compound may be suitable for treatment of virus-induced inflammatory pathologies that lead to tissue damage.

We found that PPS treatment protected PR8-infected mice from acute weight loss over the course of infection, and our PPS treatment may improve blood oxygen saturation (%SpO2) during acute PR8 infection. Interestingly, PPS-treated mice did exhibit disease signs (weight loss) compared to uninfected mice, albeit significantly less than vehicle-treated mice.

Early inhibition of viral attachment and entry represents a key advantage in acute disease management, and several antiviral drugs targeting these mechanisms have been developed and tested (10, 41, 42). PPS has been shown to inhibit human T-lymphotropic virus 1 (HTLV-1) replication *in vitro* (43), and recent studies indicate that PPS inhibits SARS-CoV-2 infection *in vitro* by modifying the receptor binding domain of S1 protein (44, 45). We found that at 8 dpi, viral titers in the lungs of mice infected with PR8 were not affected by PPS treatment, with no differences observed between infected mice treated with vehicle or with 3 mg/kg PPS. These findings suggest that the effect of PPS on weight loss and %SpO_2_ is independent of viral replication in the lungs.

Despite clear indications that PPS treatment helped reduce disease severity, histological analysis of lung sections did not show any significant changes in infiltration or overall pathology. However, flow cytometry analysis showed that rather than reducing overall inflammatory infiltrates in the lungs, PPS treatment was associated with the retention of alveolar macrophages, and a reduction in the proportion of CCR2+ inflammatory monocytes in the lungs of mice treated with 3 and 6 mg/kg PPS. Alveolar macrophages are a yolk sac-derived subset of tissue-resident macrophage which establishes early during development, and provides a first-line of defense against airway pathogens (46, 47). Alveolar macrophages act both by clearing airway pathogens *via* phagocytosis, or efferocytosis of apoptotic cells, and by limiting excessive inflammation through the release of anti-inflammatory factors, including transforming growth factor (TGFβ), Prostaglandin E2 (PGE2) and platelet-activating factor (PAF) into the tissue microenvironment (48, 49). These macrophages have been shown to be critical for protection against IAV infection (50, 51) and other respiratory pathogens such as Respiratory syncytial virus (RSV) and Streptococcus pneumoniae (49). Our results indicate that PPS may exert a protective effect in the lung that enables alveolar macrophages to persist and potentially assist in limiting tissue damage.

Although we did not observe a significant change in the number of T cells in the lungs, the proportion of CD4+ (but not CD8+) T cells was significantly higher in the lungs of mice treated with 3 mg/kg PPS, and higher (though not statistically significant) in the lungs of mice treated with 6 mg/kg PPS, suggesting that a reduced inflammatory milieu may facilitate the expansion of lung CD4+ T cells subsets, which may include helper type-1 CD4+ (Th1) T cells. Interestingly, while our observations indicated that alveolar macrophages were likely retained in the lungs of mice treated with PPS, other myeloid cell subsets were not significantly affected by PPS treatment.

The impact of pro-inflammatory chemokines and cytokines was investigated, be ELISA, and although we did not consistently observe decreased levels across all experimental repeats, some independent experiments showed clear evidence of reductions in CCL2, IL-6, IFN-γ and IL-12p70 in the serum of mice treated with PPS. Interestingly, only CCL2 serum levels appeared to be reduced in mice treated with both 3 and 6 mg/kg PPS, while IL-6, IFN-γ and IL-12p70 serum levels were only reduced in mice treated with 6 mg/kg PPS, compared to vehicle-treated mice. Of note, we did not observe any decreases in these markers in the lung tissue of mice treated with PPS, with the exception of IL-12p70, which was significantly reduced in the lungs of mice treated with both 3 and 6 mg/kg in one out of three experiments. High IL-6 expression is characteristic of PR8 infection, both in mice and humans (52, 53), and do contribute to the severity of systemic pro-inflammatory responses; although the IL6-IL-6R signaling pathway is required to enable neutrophil-mediated clearance of PR8 in the lungs (54), and a lack of IL-6 signaling in mice was shown to be detrimental in PR8 infection (55, 56). Our findings that IL-6 serum levels may be affected by higher PPS doses may account for the disease phenotype we observed in PPS-treated mice.

CCL2, which drives macrophage differentiation during inflammatory response (57) is also locally expressed in inflamed lungs and helps recruit CCR2+ monocytes from the circulation during PR8 infection (58). Although we only observed a decrease in serum CCL2 in mice treated with PPS, CCR2+ inflammatory monocytes were reduced in the lungs of mice treated with PPS. The systemic effect of PPS, administered subcutaneously, may affect serum chemokines (like CCL2, which act through a gradient) and in turn dampen recruitment of monocytes to the lungs, but our observations do not point toward direct interference with the CCL2/CCR2 axis.

The observation that CD4+ T cell expansion may be enhanced in the lungs of PPS-treated mice could be a result of an immunoregulatory effect of PPS, in particular in the context of IFN-γ. IFN-γ is a potent inflammatory and regulatory cytokine that is also elevated in the lungs following PR8 infection (59, 60), and is required for the activation of CD8+ cytotoxic and CD4+ helper T cells and also helps control neutrophilic infiltration in the lung, thereby limiting tissue damage (60). We observed a reduction in IFN-γ at 8 dpi in the serum, but not in the lungs. Therefore, it is possible that the marginal increase in CD4+ T cells we observed in the lungs of PPS-treated mice is independent of IFN-γ, as other cytokines including TGFβ, IL-12 or IL-6 – depending on the dominant helper T cell phenotype – may play a more defining role during PR8 infection, although studies have shown that at 8 dpi, Th1, TFH (follicular helper) and TREG (regulatory) T cell phenotypes, which are regulated by IFN-γ, IL-6/IL-21 and IL-10, respectively, are the main infiltrating CD4+ T cell subtypes found in the lungs of PR8-infected mice (61). Future studies should therefore dissect how PPS treatment impacts the phenotypic diversity of lung T cell subsets in PR8 infection.

One of the key outcomes of the effects of PPS was reduced weight loss in mice infected with PR8. Cachexia, mediated weight loss during disease, has been extensively described in a range of inflammatory illnesses, both acute and chronic (62). Cytokines that are most implicated with cachexia include IFN-γ, as well as IL-1β, TNF-α and IL-6 (63–65), yet only IL-6 and IFN-γ levels in the serum appeared to have been affected following PPS treatment. This indicates that, although TNF-α and IL-6 are well-accepted drivers of cachexia in disease (66, 67), our observations may be explained by the regulatory effect of PPS on NF-κB resulting in the downregulation of these cytokines, and further studies examining the transcriptional profile of cachexia-associated pathways may shed light on how PPS treatment ameliorates cachexia in PR8-infected mice.

We next asked whether the reduction in weight loss seen in PPS-treated mice during the acute phase would lead to improved post-acute pathology. Collagen production is increased after resolution of inflammation in most tissues, to aid with tissue repair – although in post-infectious pathology, this can develop into fibrosis (68). Earlier reports linked PPS with inhibition of matrix metalloproteinases (MMPs) and ADAMTS proteins (30–32) as well as FGF-2 and elastase (69, 70), all associated with the development of fibrosis. Expert histopathological scoring of lung structural changes determined that the hallmarks of fibrotic responses (e.g., consolidation, fibrosing alveolitis) in this model of PR8 infection were not clearly visible, suggesting that PR8-induced post-acute lung disease is distinct from well-characterized fibrosis models such as bleomycin-induced fibrosis models (71, 72). However, studies have reported that sICAM-1 levels in the lungs were associated with fibrosis (38, 68), and the complement system has also been shown to be relevant in fibrosis development in the lungs. C5 complement proteins were also shown to drive inflammation and neutrophil recruitment in PR8 infection in mice (73), and C5b9 and CF-Bb, which are integral components of the complement membrane attack complex, are recruited downstream of C5 activation (74, 75). PPS administered at a 6mg/kg dose either prophylactically or therapeutically showed significant reductions in lung sICAM-1 at 21 dpi in PR8-infected mice. Reduced levels of sICAM-1 in the post-acute phase suggests that PPS treatment both over early (Day 0 to Day 7) and late (Day 5 to Day 12) phases of PR8 infection may dampen post-acute lung consolidation, and this was reflected by our observations on the levels of complement proteins associated with fibrosis. Levels of C5b9 were significantly reduced in mice treated with PPS while levels of Complement Factor Bb (CF-Bb) were not affected by PPS treatment. C5b9, which triggers the complement’s membrane attack complex (MAC), was shown to be a feature of viral infection with SARS-CoV-2, as this complex is deposited in the alveolar septa of COVID-19 patients (76). While the exact role of C5b9 in PR8 infection remains poorly understood, a study showed that in a model bleomycin-induced fibrosis, mice lacking interleukin-17 (IL-17A) developed less severe fibrosis, and this was concomitant with a reduction in C5b9 deposition (35). CF-Bb is required for the induction of alternative complement pathways such as the mannan-binding lectin (MBL) pathway (77), which has been shown to exert a protective effect in PR8 infection *in vitro* (78), and this effect was dependent on alveolar macrophages. This suggests that the potential role of complement (and its associated pathways) in PR8 could be linked to the PPS-induced maintenance of alveolar macrophages we observed in this study.

In conclusion, this study investigated the potential therapeutic effects of the semi-synthetic polysaccharide, PPS in a model of lung inflammation mediated by PR8 (PR8 strain) infection. The key observations are that PPS demonstrated potential anti-inflammatory effects during the acute phase of pulmonary infection mediated by PR8 as observed by the reduction in weight loss and improvement in oxygen saturation following PPS treatment, as well as an enrichment in protective alveolar macrophages and CD4+ T cells in the lungs. In addition, the preceding anti-inflammatory actions of PPS during the acute phase may have had a role in modulating the post-acute pulmonary changes based on the observations of reduced fibrotic biomarkers.

## Data availability statement

The original contributions presented in the study are included in the article/**Supplementary material**. Further inquiries can be directed to the corresponding authors.

## Ethics statement

The animal study was reviewed and approved by Griffith University Animal Ethics Committee Griffith University, Parklands Dr, Southport QLD 4215.

## Author contributions

Conceptualisation: RK, SM, AZ. Data curation: CS, AZ. Formal analysis: AZ. Funding acquisition: SM. Investigation: AZ, HM, JF, XL. Methodology: AZ, HM, JF, XL. Project administration: SM. Supervision: SM, AZ. Visualisation: AZ, SM. Writing – original draft: AZ, SM. Writing – review and editing: RK, CS, SM, AZ. All authors contributed to the article and approved the submitted version.

## Supplementary material

Supplementary Figure 1 Effect of subcutaneous and intraperitoneal administration of PPS in PR-8 infected mice. Weight loss in uninfected mice (uninfected; Uninf), uninfected mice treated with PPS (Uninf + PPS (sc)) or vehicle (Uninf + Veh (ip)) subcutaneously (SC) or intraperitoneally (IP), PR8-infected mice treated with PPS SC (PR8 + PPS (sc)) or IP (PR8 + PPS (ip)), and PR8-infected, vehicle-treated mice SC (PR8 + Veh (sc)) or IP (PR8 + Veh (ip)). ***p<0.01, ***p<0.005* and ns: not significant for PR8 + Veh (ip) compared with PR8 + PPS (ip); *^###^p<0.005, ^####^p<0.0001* for PR8 + Veh (sc) compared with PR8 + PPS (sc). n=7 per group; n=4 for uninfected mice. Statistically significant differences were assessed by two-way ANOVA & *p* values shown on plots

Supplementary Figure 2 Pro-inflammatory cytokine/chemokine measurement in the lungs and serum of PR8-infected mice treated with PPS. Changes in lung and serum levels of CCL2, IL-6, IL-12p70, IL-10, IFN-γ and TNF-α measured by ELISA in Experiments 2 (Expt#2) and Experiment 3 (Expt#3). Values for lung concentrations were normalised to tissue weights. Values for serum concentrations shown as per mL of serum. Box-whiskers plot show all data points (max/min), standard error, median (line in box) and mean (‘+’ in box). Statistically significant differences were assessed by one-way ANOVA with a Sidak post-test *(*p<0.05*; ***p<0.01*; *****p<0.0001*; ns: not significant). (n=6 mice per group per experiment; Data shown from two of 3 independent experiments).

Supplementary Figure 3 Gating strategy used for the identification of immune cells in the lungs of PR8-infected mice. Representative plots outlining sequential gating of CD45^+^ live cells, Ly6G^+^ neutrophils, CD11b^hi^Ly6C^hi^ inflammatory monocytes (IM; derived from green panel), CD64^+^SiglecF^+^ alveolar macrophages (AM; derived from blue panel) and CD4^+^ and CD8^+^ T cells (derived from pink square).
