# Supplementary figures and images for: Anti-inflammatory actions of Pentosan polysulfate sodium in a mouse model of influenza virus A/PR8/34-induced pulmonary inflammation
Source: Front Immunol. 2023 Feb 9;14:1030879. doi: 10.3389/fimmu.2023.1030879 (PMC9947849; doi:10.3389/fimmu.2023.1030879)

# Supplemental Figure 1

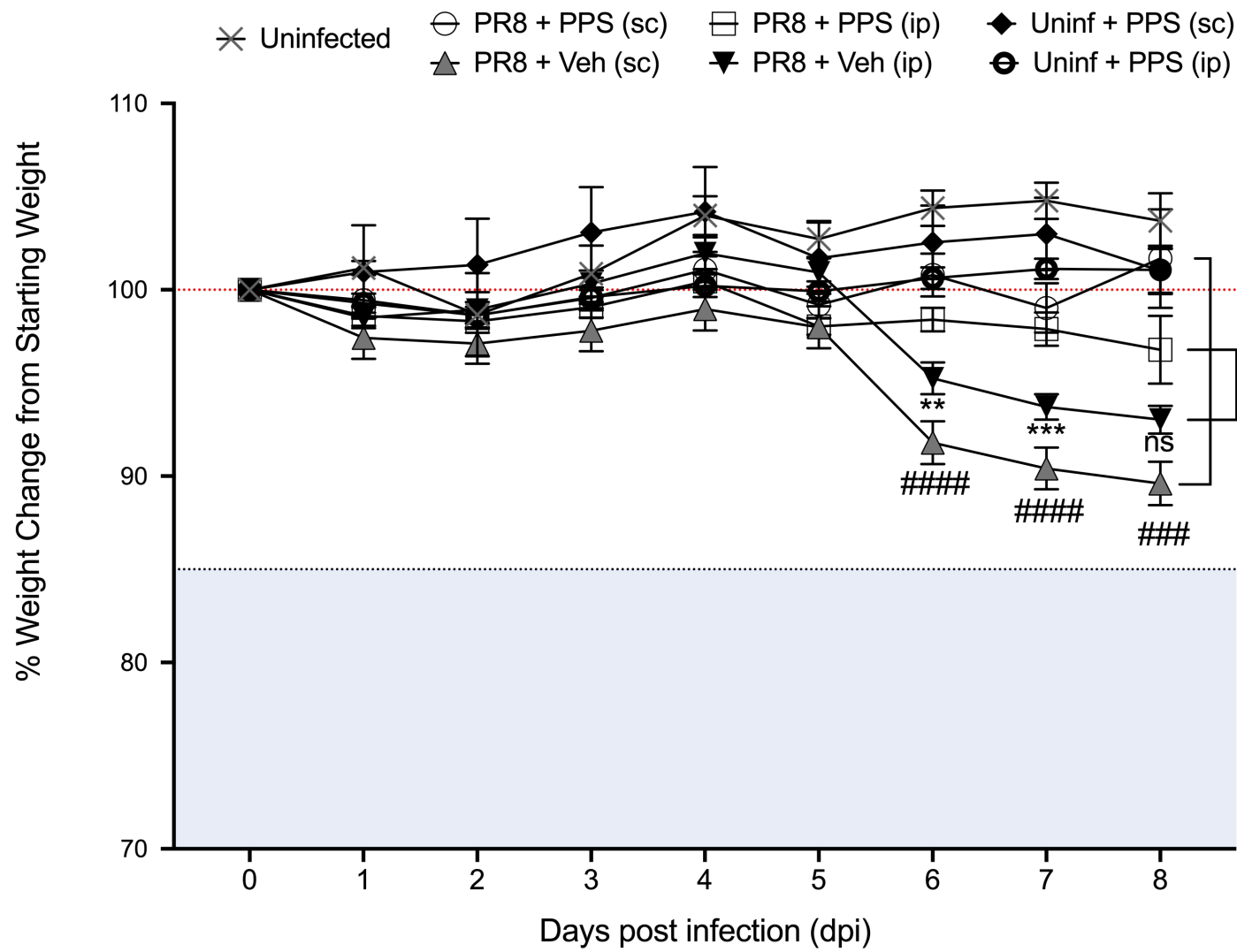

Supplement: Supplementary Figure 1 — Effect of subcutaneous and intraperitoneal administration of PPS in PR-8 infected mice. Weight loss in uninfected mice (uninfected; Uninf), uninfected mice treated with PPS (Uninf + PPS (sc)) or vehicle (Uninf + Veh (ip)) subcutaneously (SC) or intraperitoneally (IP), PR8-infected mice treated with PPS SC (PR8 + PPS (sc)) or IP (PR8 + PPS (ip)), and PR8-infected, vehicle-treated mice SC (PR8 + Veh (sc)) or IP (PR8 + Veh (ip)). **p<0.01, ***p<0.005 and ns: not significant for PR8 + Veh (ip) compared with PR8 + PPS (ip); ###p<0.005, ####p<0.0001 for PR8 + Veh (sc) compared with PR8 + PPS (sc). n=7 per group; n=4 for uninfected mice. Statistically significant differences were assessed by two-way ANOVA & p values shown on plots [file Image_1.pdf]

## Supplemental Figure 2

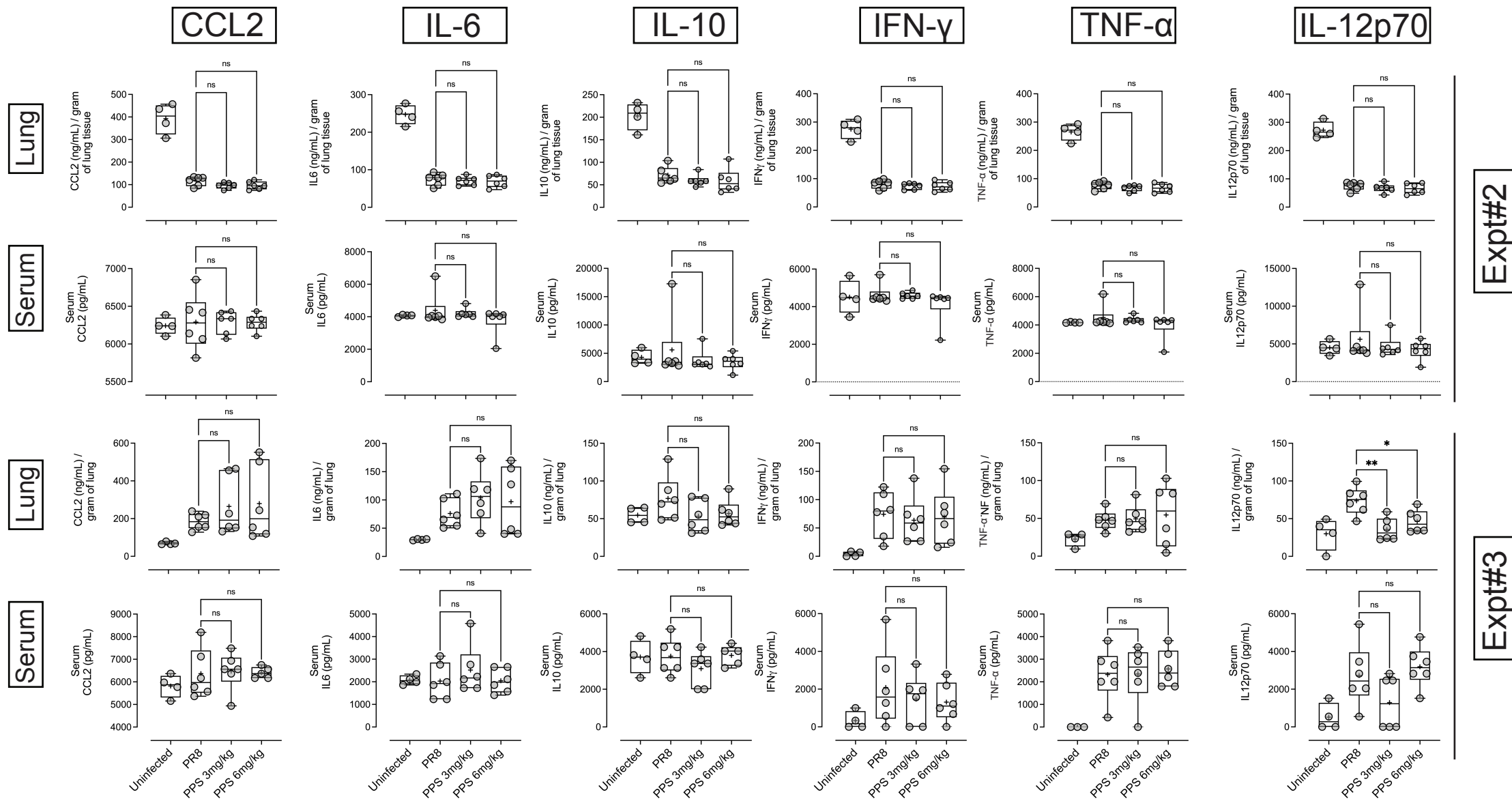

Supplement: Supplementary Figure 2 — Pro-inflammatory cytokine/chemokine measurement in the lungs and serum of PR8-infected mice treated with PPS. Changes in lung and serum levels of CCL2, IL-6, IL-12p70, IL-10, IFN-γ and TNF-α measured by ELISA in Experiments 2 (Expt#2) and Experiment 3 (Expt#3). Values for lung concentrations were normalised to tissue weights. Values for serum concentrations shown as per mL of serum. Box-whiskers plot show all data points (max/min), standard error, median (line in box) and mean (‘+’ in box). Statistically significant differences were assessed by one-way ANOVA with a Sidak post-test (*p<0.05; **p<0.01; ****p<0.0001; ns: not significant). (n=6 mice per group per experiment; Data shown from two of 3 independent experiments). [file Image_2.pdf]

# Supplemental Figure 3

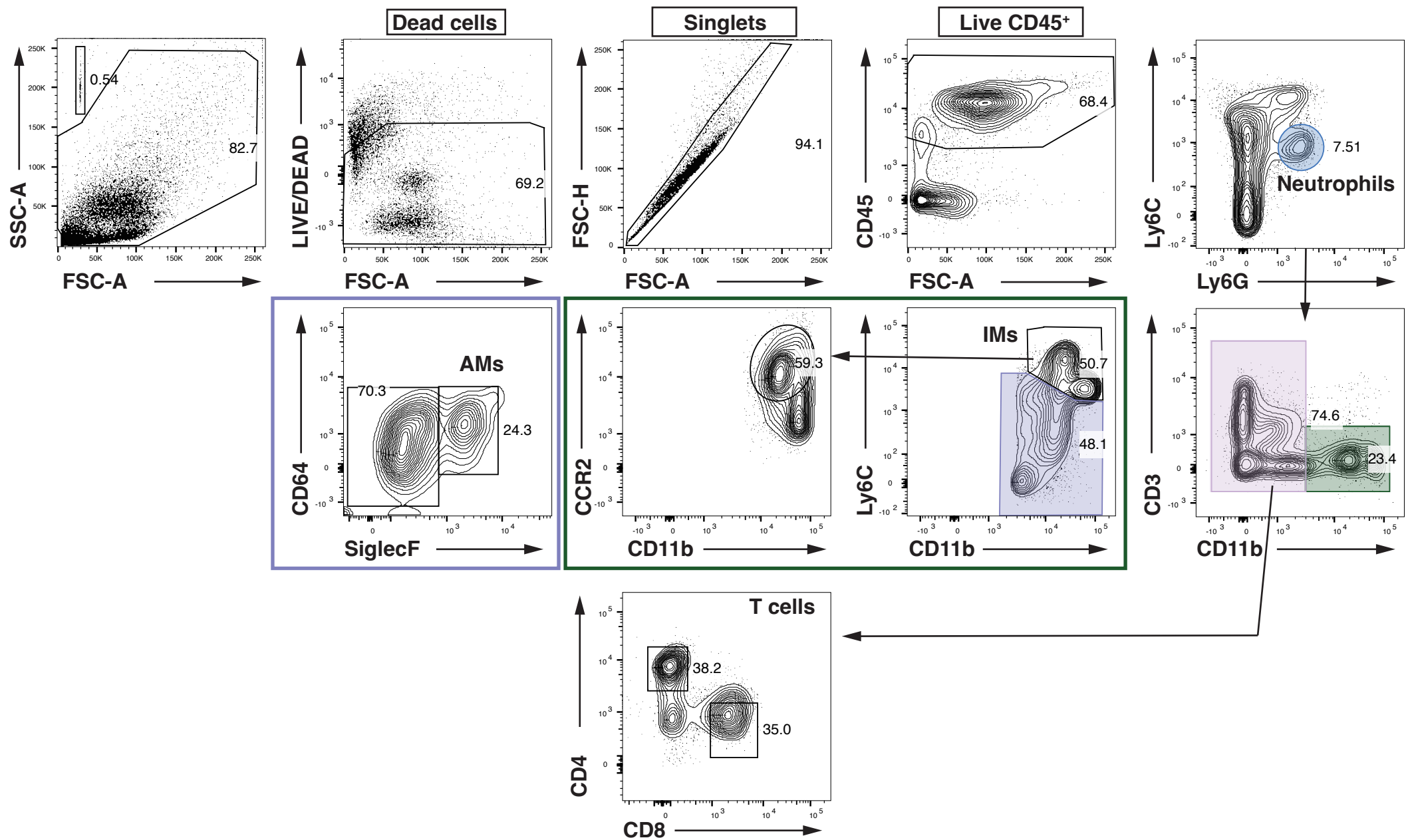

Supplement: Supplementary Figure 3 — Gating strategy used for the identification of immune cells in the lungs of PR8-infected mice. Representative plots outlining sequential gating of CD45+ live cells, Ly6G+ neutrophils, CD11bhiLy6Chi inflammatory monocytes (IM; derived from green panel), CD64+SiglecF+ alveolar macrophages (AM; derived from blue panel) and CD4+ and CD8+ T cells (derived from pink square). [file Image_3.pdf]
